# Effectiveness of eHealth Interventions for HIV Prevention and Management in Sub-Saharan Africa: Systematic Review and Meta-analyses

**DOI:** 10.1007/s10461-021-03402-w

**Published:** 2021-08-24

**Authors:** Louisa Manby, Catherine Aicken, Marine Delgrange, Julia V. Bailey

**Affiliations:** 1 Institute of Epidemiology and Healthcare, University College London, London, UK; 2 School of Health Sciences, University of Brighton, Falmer, Brighton, UK; 3 Centre for Population Research in Sexual Health and HIV, Institute for Global Health, University College London, London, UK; 4 eHealth Unit, Department of Primary Care and Population Health, University College London (Royal Free Campus), Rowland Hill Street, London, NW3 2PF UK

**Keywords:** Human immunodeficiency virus, eHealth, Sub-Saharan Africa, Systematic review, Meta-analysis

## Abstract

HIV is still the leading cause of death in Sub-Saharan Africa (SSA), despite medical advances. eHealth interventions are effective for HIV prevention and management, but it is unclear whether this can be generalised to resource-poor settings. This systematic review aimed to establish the effectiveness of eHealth interventions in SSA. Six electronic databases were screened to identify randomised controlled trials (RCTs) published between 2000 and 2020. Meta-analyses were performed, following Cochrane methodology, to assess the impact of eHealth interventions on HIV-related behaviours and biological outcomes. 25 RCTs were included in the review. Meta-analyses show that eHealth interventions significantly improved HIV management behaviours (OR 1.21; 95% CI 1.05–1.40; Z = 2.67; p = 0.008), but not HIV prevention behaviours (OR 1.02; 95% CI 0.78–1.34; Z = 0.17; p = 0.86) or biological outcomes (OR 1.17; 95% CI 0.89–1.54; Z = 1.10; p = 0.27) compared with minimal intervention control groups. It is a hugely important finding that eHealth interventions can improve HIV management behaviours as this is a low-cost way of improving HIV outcomes and reducing the spread of HIV in SSA. PROSPERO registration number: CRD42020186025.

**Supplementary Information:**

The online version contains supplementary material available at 10.1007/s10461-021-03402-w.

## Background

Around 70% of global HIV diagnoses are in Sub-Saharan Africa (SSA), where sexual transmission remains the primary mode of HIV transmission [1]. Despite recent medical advances, HIV is still the leading cause of death in the region [1, 2]. Poor outcomes among those living with HIV in SSA are linked to delayed diagnosis, poor linkage to HIV care and inconsistent adherence to anti-retroviral therapies [3]. More than half of people living with HIV in SSA are unaware of their HIV status and ~ 60% are not receiving treatment [4]. Given SSA's high disease burden and extreme shortage of healthcare workers, there remains an unmet need for interventions which deliver tailored support for the prevention and management of sexually transmitted HIV in the region and digital health strategies have great potential to meet this need [5].

Information and communications technology use has been rapidly increasing since the early 2000s in SSA [6, 7]. The majority of the SSA population own a mobile phone and ownership is now as high as 80% in some countries [8]. Basic phones are the most common mobile device owned in SSA and the likelihood of ownership is related to income and level of education [8]. With increasing technology use, eHealth in SSA has expanded over the last ~ 20 years and there is great enthusiasm for its potential for improving health outcomes [9, 10]. Improving HIV/AIDS outcomes has been a major focus of digital health strategies in SSA [11]. These strategies include mobile phone-based reminders and messages aimed at improving ART medication adherence and attendance to medical appointments as well as providing health information related to HIV treatment and prevention [11].

Systematic reviews have demonstrated the effectiveness of eHealth interventions for HIV prevention and management [12–14], but it is unclear whether findings can be generalised to resource-poor settings [9, 10]. As such, this study aimed to systematically evaluate the effectiveness of eHealth interventions for sexually transmitted HIV prevention in SSA.

## Methods

The protocol for this review was registered on PROSPERO (Registration Number CRD42020186025). This systematic review aimed to combine the results of randomised controlled trials (RCTs) to determine the effectiveness of eHealth interventions for HIV prevention and management in SSA, in comparison to control groups. This review also aimed to compare the effectiveness of interactive and non-interactive eHealth interventions for HIV prevention and management in SSA.

The Theory of Planned Behaviour explains how changes in cognitive outcomes can drive changes in behaviours which in turn can drive improvements in health outcomes [15]. Based on this theory, we decided to use behavioural and biological outcomes as primary measures to determine the effectiveness of interventions, with cognitive and other outcomes as secondary measures.

### Inclusion Criteria

*Participants* eligible studies were those conducted in SSA countries, as defined by the World Bank [16].

*Interventions* eHealth interventions were defined as those which were accessible through mobile/Internet/digital means for the purpose of education or behaviour change related to the prevention of sexually transmitted HIV. Interventions could be for the target populations themselves, or sexual health educators (e.g., healthcare workers, community leaders) provided outcomes were measured among the target population. Given that in low-resource settings access to ICT may be limited, we felt that sexual health educators could play a facilitative role in delivering eHealth interventions for HIV prevention and management.

Interventions could be interactive or non-interactive. eHealth interventions were classified as “interactive” when they allowed two-way communication with the user. Interactive interventions required either an automated, programmed system or an active human component to respond to user input.

*Study designs* randomised controlled trial (RCT) studies published in English from the year 2000.

*Comparators* minimal intervention (e.g., standard care only) or face-to-face intervention.

*Outcomes* outcomes were categorised as cognitive, behavioural (HIV prevention or HIV management), biological, or other outcomes related to HIV acquisition. Cognitive outcomes included HIV related knowledge, perceived HIV-related stigma, perceived severity of and susceptibility to HIV, self-efficacy (an individual’s belief in their own capacity to carry out a behaviour), and intention to carry out HIV prevention behaviours. Prevention-related behavioural outcomes included condom use, HIV testing and counselling (HTC), and voluntary male circumcision. HIV management-related behavioural outcomes included HIV medication adherence, and uptake and retention in healthcare services for HIV care. Biological outcomes included HIV acquisition, HIV viral load, acquisition of opportunistic infections, and acquisition of other sexually transmitted infections. Other outcomes linked to HIV vulnerability were also included such as use of alcohol or drugs and experience of violence or abuse.

### Exclusion Criteria

We excluded studies which aimed to improve condom use for the purpose of contraception rather than STI prevention, studies of HIV prevention in the context of vertical transmission, studies with composite interventions which inextricably combined digital health interventions with other non-digital interventions, studies with digital interventions for health care workers aimed at optimising healthcare delivery and studies with digital data collection/remote monitoring interventions with no education or behaviour change components.

### Search Strategy

The search focused on eHealth interventions for education or behaviour change aimed at preventing the sexual transmission of HIV in SSA. The following databases were searched on the 5 June 2020 using a tailored search strategy for each: Medline, Embase, Web of Science, Pan-African Clinical Trials Registry, Cochrane Library, PsycInfo. Search strategies for all databases are listed in Supplementary Material 1. The search terms fell into three concepts: “HIV”, “eHealth interventions”, “Sub-Saharan Africa”. The core literature was in the intersection between the three concepts to include eHealth technologies for HIV prevention in SSA.

### Screening

Papers were screened using Mendeley software and duplicate items were removed using Mendeley’s de-duplication tool. One researcher screened the titles, abstracts and full texts for eligibility. 20% of all papers screened by title and abstract and all papers screened by full text were reviewed by a second researcher. A third reviewer was consulted, where necessary, to reach a consensus. Eligibility was determined based on pre-defined criteria for inclusion and exclusion.

### Data Extraction

One researcher recorded author names, study locations and main findings from the included papers in Excel. All extracted data were independently reviewed by a second researcher to ensure all relevant information was captured accurately.

### Data Analysis

Meta-analyses were undertaken in Cochrane’s Review Manager software version 5.4. Conceptually similar outcomes were grouped into the following four categories for analysis: HIV-related cognitive outcomes, HIV prevention behaviours, HIV management behaviours, and biological outcomes. We grouped diverse outcomes into conceptually similar groups, in order to evaluate the effect of eHealth interventions on the outcomes targeted by specific interventions.

When multiple outcomes within the same conceptual group were reported in any one study, one outcome was selected to be included in the analysis. Outcomes were prioritised for selection according to the following criteria: the study’s primary outcome, outcomes related to the key aim of the intervention, HIV testing outcomes, condom-use outcomes, objectively measured outcomes, and findings from the longest follow-up period. We sought to evaluate the effect of eHealth interventions on biological outcomes, via their ability to change behaviours related to HIV prevention and management. Behavioural and biological outcomes were therefore the main (primary) outcomes of interest in this review, and these findings were meta-analysed. Findings relating to precursors of behaviour change (cognitive and other outcomes) are also presented.

For meta-analysis, odds ratios (OR) were calculated where possible for each included outcome using the number of events in the intervention and control arms. In some instances means and standard deviations were reported, and standardised mean differences were calculated and re-expressed as ORs following guidance in the Cochrane Handbook [17]. The generic inverse variance method was used to include such data. Where data were not reported for the selected outcome measure, authors were contacted. ORs in each outcome category were then pooled using a random-effects model, which allows outcomes measured using different scales to be combined. Sub-group analyses were undertaken to determine any differences in the effectiveness of interactive (with and without an active human component) and non-interactive eHealth interventions. The characteristics used for sub-group analysis were specified in advance. The Higgins I^2^ statistic was used to assess heterogeneity. An I^2^ statistic of 0%, 1–30%, 31–50% and > 50% indicated no, minimal, moderate and substantial heterogeneity respectively [17]. All calculations were checked by a second researcher.

### Risk of Bias and Quality Assessment

The Cochrane Risk of Bias Tool was used to determine risk of bias in the RCTs. Where there was disagreement between the two researchers who independently reviewed screening, data extraction, and calculations, this was resolved by consulting a third researcher. Studies with retention > 80% were classed as having low risk of attrition bias. Where meta-analyses contained > 10 studies, funnel plot asymmetry was used to assess the risk of publication bias across included RCTs [18].

## Results

**Fig. 1:**
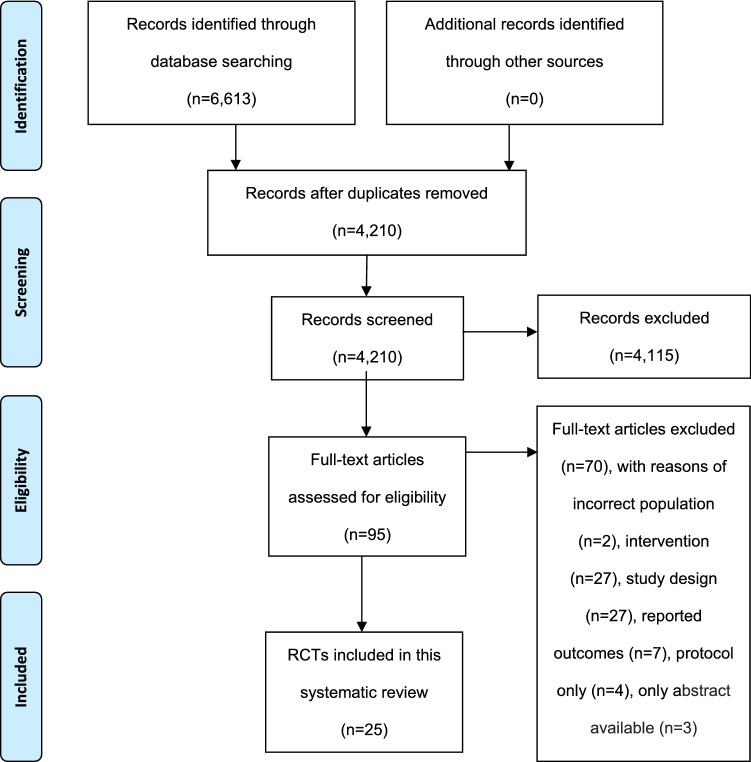
PRISMA flow diagram [19]

A total of 4210 unique records were screened by title and abstract, and full texts of 95 potentially eligible papers were screened, resulting in 25 studies being included (see PRISMA flow diagram, Fig. 1) [19].

### Characteristics of Included Studies

Study characteristics of the 25 RCTs included are summarised in Table 1. Studies were undertaken in 10 of the 48 SSA countries, with 8 in Kenya, 7 in Uganda and 5 in South Africa [16]. 14 Studies evaluated interactive eHealth interventions and 12 studies evaluated non-interactive eHealth interventions. *Non-interactive eHealth interventions* included one-way SMS providing HIV-related information as well as motivational messages and appointment reminders (see intervention characteristics in Supplementary Material 2). *Interactive eHealth interventions without an active human component* included smartphone games and internet-based programmes, whilst *interactive eHealth interventions with an active human component* included two-way SMS and mobile phone delivered counselling services. Interventions were delivered alongside standard care.

**Table 1:** Characteristics of included studies

Study author, year	Country	Intervention type	Follow up	Mean participant age (years)	Sample characteristics (size)	Outcomes included in this review
Barnabas, 2016 [20]	South Africa, Uganda	SMS reminders with phone call^a^	3 months	Not given	HIV-negative uncircumcised men (518)	Uptake of medical male circumcision
De Tolly, 2011 [21]	South Africa	One-way SMS^a^	3 weeks	Not given	General population (2553)	Uptake of HIV testing and counselling
Govender, 2019 [22]	South Africa, Zimbabwe, Mozambique	One-way SMS^a^	6 months	Not given	Truck drivers and sex workers (1783)	Uptake of HIV testing; HIV related knowledge, self-efficacy and attitudes
Haberer, 2016 [23]	Uganda	One-way SMS^a^	9 months	Not given	HIV-positive adults (62)	Adherence to ART; viral suppression
Harder, 2019 [24]	Kenya	Phone-delivered motivational interviewing^a^	6 months	38	Adults with alcohol use problems (300)	Alcohol use
Haruna, 2018 [25]	Tanzania	Sexual health game-based programme	1 week	14.1	Adolescent school students (120)	HIV-related knowledge
Joseph Davey, 2016 [26]	Mozambique	One-way SMS^a^	12 months	Not given	HIV-positive adults (830)	Retention in ART care
Kalichman, 2019 [27]	South Africa	Phone delivered counselling sessions^a^	2 weeks	34	HIV-positive patients (50)	Adherence to ART; HIV related attitudes
Kiwanuka, 2018 [28]	Uganda	SMS with phone call^b^	18 months	Not given	HIV-negative persons (662)	Retention in HIV vaccine trial
Kurth, 2019 [29]	Kenya	Internet-based counselling programme^a^	9 months	37.5	HIV-positive adults (236)	Adherence to ART; HIV viral suppression
Lapinski, 2008 [30]	Nigeria	Film about HIV-related stigma^a^	Immediate	27.6	General population (100)	HIV related attitudes
Leiby, 2016 [31]	Zambia	One-way SMS^a^	6 months	Not given	Uncircumcised men (1652)	Uptake of medical male circumcision
Lester, 2010 [32]	Kenya	Two-way SMS with phone call^a^	12 months	36.7	HIV-positive adults (538)	Adherence to ART; HIV viral suppression
Linnemayr, 2017 [33]	Uganda	One- and two-way SMS^a^	12 months	18.3	HIV-positive youth (332)	Adherence to ART
MacCarthy, 2020 [34]	Uganda	One-way SMS^a^	36 weeks	Not given	HIV-positive youth (179)	Adherence to ART
Mbuagbaw, 2012 [35]	Cameroon	SMS with phone call^a^	6 months	40.2	HIV-positive adults (200)	Adherence to ART; absence of opportunistic infections
Nsagha, 2016 [36]	Cameroon	One-way SMS^a^	1 month	38.8	HIV-positive adults (90)	Adherence to ART
Odeny, 2014 [37]	Kenya	One-way SMS^a^	42 days	Not given	Adult men who had undergone male circumcision (1200)	Abstinence from sex post circumcision
Pop-Eleches, 2011 [38]	Kenya	One-way SMS^a^	48 weeks	36.3	HIV-positive adults (428)	Adherence to ART
Reid, 2017 [39]	Botswana	One-way SMS^a^	6 months	41.1	HIV-positive adults (108)	ART pharmacy visit attendance; CD4 cell count
Van der Kop, 2018 [40]	Kenya	SMS with phone call^a^	14 months	33.7	HIV-positive adults (700)	Retention in HIV care
Venter, 2019 [41]	South Africa	Educational app with reminders^a^	8 months	Not given	HIV-positive adults (353)	Linkage to care for HIV; HIV viral suppression
Winskell, 2018 [42]	Kenya	Smartphone game^a^	6 weeks	12.7	Pre-adolescent school students (60)	HIV related knowledge, self-efficacy, attitudes, and intentions
Ybarra, 2013 [43]	Uganda	Internet-based sexual health programme^a^	6 months	16.1	Secondary school students (366)	Condom use
Ybarra, 2015 [44]	Uganda	Internet-based sexual health programme^a^	6 months	16.1	Adolescent school students (366)	HIV related knowledge, self-efficacy, attitudes, and intentions

^a^Minimal intervention control

^b^Face-to-face control

Control groups received minimal interventions in 24 RCTs (i.e., e.g., standard care only). One RCT compared an eHealth intervention with a face-to-face comparator in which participants received in-person reminders to improve retention in an HIV vaccine efficacy trial.

Follow-up data collection ranged from immediate to 18 months and sample size ranged from 50 to 1783. Over half of the studies included (n = 13) were conducted solely among people living with HIV. 4 Studies targeted school students and 2 studies targeted high-incidence populations (one among truck drivers and sex workers and the other among people with alcohol use problems).

### Quality Assessment

Table 2 summarises the quality of the 25 RCTs determined using the Cochrane Risk of Bias tool. 17 of the studies were judged “low risk of bias” regarding random sequence generation and 10 regarding allocation concealment. 22 Studies either did not report blinding of participants/personnel or were unblinded; however, given the nature of the interventions this was unsurprising. 19 of the 25 studies had low risk of attrition bias. 5 Studies were at risk of reporting bias as not all outcomes appeared to be analysed and reported in line with a pre-specified plan.

**Table 2:**
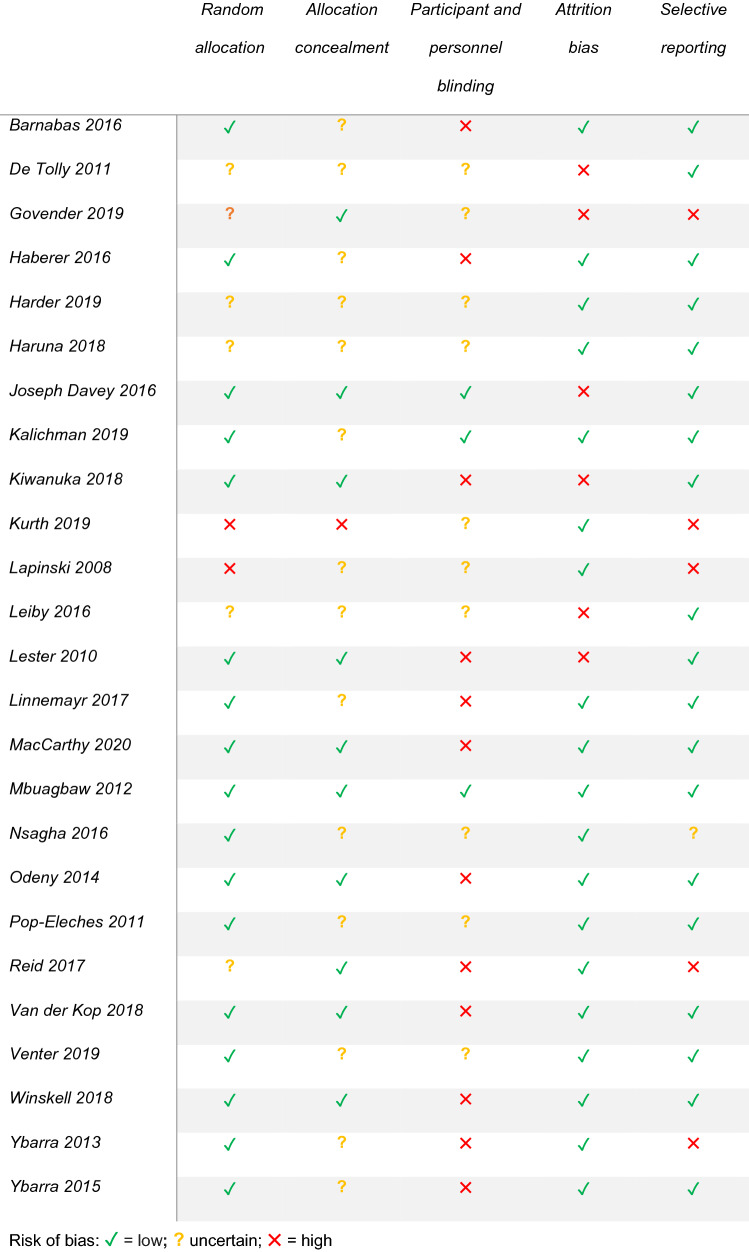
Quality appraisal of the studies

### Effectiveness of eHealth Interventions for HIV Prevention and Management

We included data from 25 RCTs, which randomised a total of 15,343 participants: 2356 were randomised to interactive interventions, 5530 to non-interactive interventions and 5808 to controls.

#### Can eHealth Technologies Improve HIV Prevention Behaviours?

**Fig. 2:**
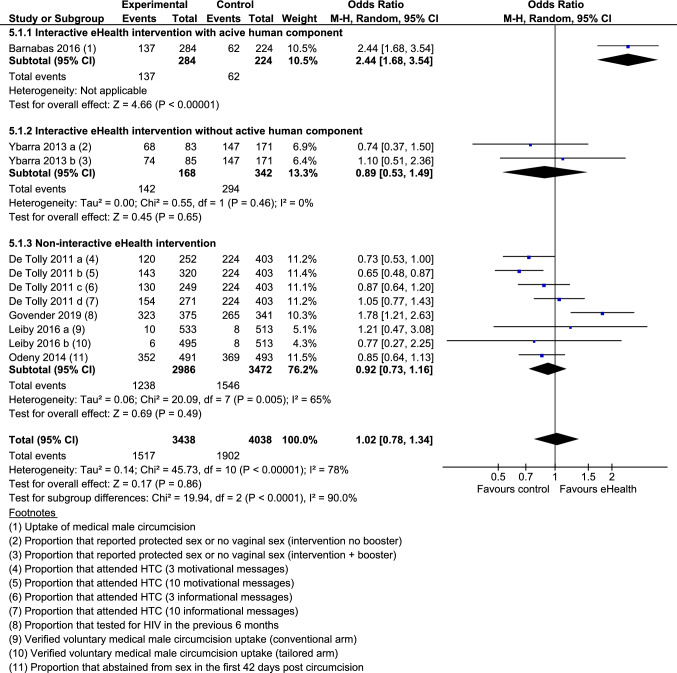
Forest plot: behaviours related to HIV prevention

6 Studies reported outcomes related to HIV prevention behaviours [20–22, 31, 37, 43]. Behaviours included condom use, attendance for HIV testing and counselling, and uptake of medical male circumcision. Meta-analysis showed that the odds of engaging in HIV prevention behaviours were not significantly higher among participants who received an eHealth intervention compared with minimal intervention control (OR 1.02; 95% CI 0.78–1.34; Z = 0.17; p = 0.86) (Fig. 2). Overall, substantial heterogeneity was present (I^2^ = 78%; χ^2^ = 45.73; p < 0.00001).

Sub-group analysis demonstrated that one interactive eHealth intervention with an active human component significantly improved HIV prevention behaviours whereas eHealth interventions without an active human component (2 study arms) and non-interactive eHealth interventions (8 study arms) did not. Significant sub-group differences were found (χ^2^ = 19.94; p < 0.0001).

#### Can eHealth Technologies Improve HIV Management Behaviours?

**Fig. 3:**
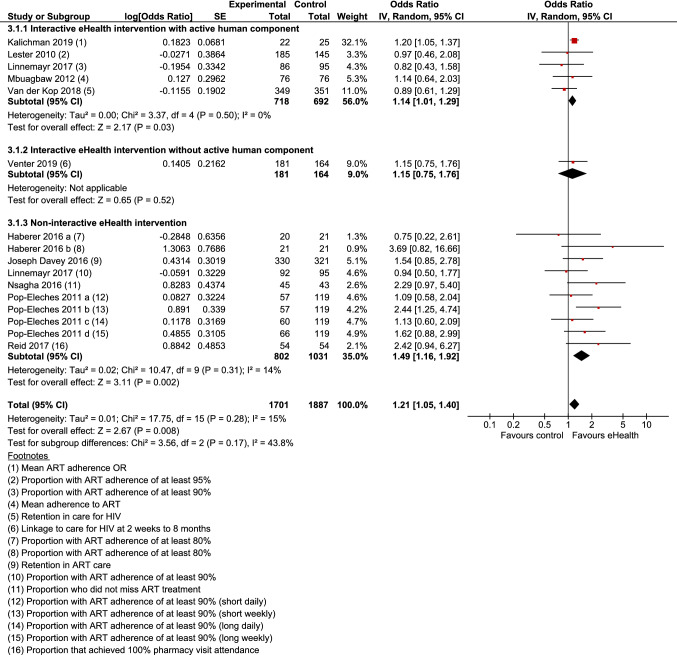
Forest plot: behaviours related to HIV management

13 Studies reported impacts on behavioural outcomes related to the management of HIV and compared eHealth interventions with minimal intervention control groups. Behavioural outcomes included adherence to ART, attendance at pharmacy visits, and linkage to and retention in HIV care. Two of these studies reported no significant differences in behaviour between study arms, but the data provided in these papers was not in a suitable format for analysis and was not provided by authors upon request [29, 34]. Meta-analysis of 11 studies [23, 26, 27, 32, 33, 35, 36, 38–41] showed that the odds of engaging in behaviours for HIV management were 21% higher among those in the intervention group compared with control (OR 1.21; 95% CI 1.05–1.40; Z = 2.67; p = 0.008) (Fig. 3). Overall, minimal heterogeneity was present (I^2^ = 15%; χ^2^ = 17.75; p = 0.28). There was no evidence of funnel plot asymmetry, indicating a low risk of publication bias across these studies.

In sub-group analysis, interactive eHealth interventions with active human components (5 studies) and non-interactive eHealth interventions (6 studies) led to a statistically significant improvement in HIV management behaviours, but an interactive eHealth intervention without an active human component (1 study) did not. Overall, however, sub-group differences were not statistically significant (χ^2^ = 3.56; p = 0.17).

One RCT was not included in meta-analyses as it was the only study that had a face-to-face control [28]. This study reported significantly worse study retention in the eHealth intervention arm (where participants received short SMS and phone call visit reminders) compared with control (where participants received face-to-face visit reminders) (p = 0.021).

#### Can eHealth Technologies Improve HIV-Related Biological Outcomes?

**Fig. 4:**
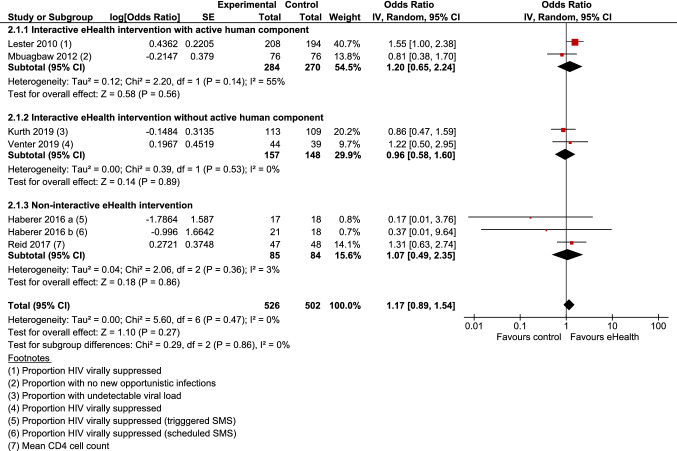
Forest plot: HIV-related biological outcomes

6 Studies reported HIV-related biological outcomes [23, 29, 32, 35, 39, 41]. Outcomes included proportion of participants virally suppressed and with no new opportunistic infections, and mean CD4 cell count. Meta-analysis showed that the odds of improved biological outcomes were not significantly higher among those who received an eHealth intervention compared with minimal intervention control (OR 1.17; 95% CI 0.89–1.54; Z = 1.10; p = 0.27) (Fig. 4). Overall, no heterogeneity was present (I^2^ = 0%; χ^2^ = 5.60; p = 0.47).

Sub-group analysis demonstrated that compared with minimal intervention controls the impact of eHealth interventions on HIV-related biological outcomes was not significant for any of the three sub-groups: interactive eHealth interventions with an active human component (2 studies), interactive eHealth interventions without an active human component (2 studies) and non-interactive eHealth interventions (2 studies). Overall, sub-group differences were not statistically significant (χ^2^ = 0.29; p = 0.86).

#### Can eHealth Technologies Improve HIV-Related Cognitive and Other Outcomes?

Cognitive outcomes reported were HIV-related knowledge, self-efficacy, attitudes and intentions. All the 4 studies that reported HIV-related knowledge found that eHealth interventions significantly improved knowledge compared with control [22, 25, 42, 44]. 3 Studies measured self-efficacy: 1 study reported significantly improved self-efficacy [42] and 2 reported no statistically significant differences between intervention and control groups [22, 44]. 5 Studies measured attitudes (towards condom use, towards the perceived severity of and susceptibility to HIV, and towards HIV-related stigma); 2 found significant improvements in attitudes [27, 44] and 3 found no statistically significant differences between intervention and control arms [22, 30, 42]. 2 Studies measured intention to carry out HIV prevention behaviours; both found significant improvement among those in the eHealth intervention arm compared with control [42, 44]. 1 Study assessed the impact of eHealth interventions on other outcomes which found that alcohol use was significantly reduced among participants in the intervention arm compared to control [24].

## Discussion

We found that eHealth interventions deployed in SSA increased engagement in HIV management behaviours but did not significantly impact HIV prevention behaviours or HIV-related biological outcomes. eHealth interventions had positive effects on HIV-related knowledge and intentions to reduce the likelihood of, or avoid, HIV transmission/acquisition. This review provides good evidence supporting the use of eHealth interventions to improve HIV management behaviours in SSA.

The findings of this systematic review resonate with existing literature. Two other systematic reviews, which include studies from Europe, North America, South America, Africa, Oceania, and Asia, demonstrate the positive impact of eHealth interventions on HIV management behaviours. The first demonstrates the impact of eHealth interventions, predominantly SMS interventions, on adherence to ART and clinic attendance [13]. The second, larger review found similar positive effects, reporting that the odds of improved adherence to ART were more than double (OR; 95% CI = 2.15; 1.18–3.91) among those who received eHealth interventions compared with control [14]. One qualitative study undertaken in Uganda emphasised the importance of developing new habits to augment the impact of eHealth interventions on adherence to ART after intervention withdrawal [45].

We found that eHealth interventions did not significantly improve behaviours related to HIV prevention. This finding is discordant with that of Kemp and Velloza, whose 2018 review found that eHealth interventions can effectively improve uptake of HIV testing [13]. The moderate to significant heterogeneity found among the studies in our meta-analyses indicates that there was large variability in the effect sizes of the individual RCTs, which creates uncertainty in our results. Heterogeneity may have arisen due to differences between studies in participant characteristics/settings or methodologies [17].

Despite improving HIV management behaviours, our review found no impact on biological outcomes, unlike other global research which demonstrates a significant impact of mHealth on HIV viral load and CD4 cell count [46]. It is possible that this was because the overall sample size was not large enough to detect significant differences between arms. An important consideration is that changes in HIV-related biological outcomes are often difficult to measure due to the infrequency of events such as HIV acquisition (particularly when studies only have short follow-up periods) and the practical challenges of biological outcome measurement [47].

It is important to capture any differences between interactive and non-interactive interventions as there can be cost implications for interventions which require a greater level of provider support, particularly for those with an active human component [48]. Although significant sub-group differences were present in the analysis of HIV prevention behaviours, there were no statistically significant differences found between sub-groups for HIV management behaviours and biological outcomes. These results seem to contradict existing literature which reports that interactive digital interventions tend to have a greater impact than those that are non-interactive [12, 47, 49]. We know, however, that the rate of uptake of new technologies has been slower in SSA due to poorer digital infrastructure and that smartphone ownership in SSA is lower than any other region worldwide [8], which may explain why interactive Internet-based sexual health programmes and smartphone apps/games are not as effective as we would expect based on the findings from other studies.

### Strengths and Limitations

This systematic review is the first to investigate the effectiveness of eHealth interventions for HIV prevention and management in SSA. We included 25 RCTs which are the most appropriate design to assess effectiveness [50]. Included studies were conducted in 10 of SSA’s 48 countries, which is likely to reflect differences in HIV research activity within the region, although it is also possible that we missed relevant papers by restricting our review to English language publications. Very few of the included studies were at low risk of any bias, and some study data could not be obtained for inclusion in meta-analyses.

### Recommendations for Future Work

More RCTs on eHealth interventions for HIV prevention and management in SSA are required to provide greater certainty in the evidence of their effectiveness, including measurement of biological outcomes. More qualitative studies are also needed to understand the context and complexity of health needs, and the feasibility and acceptability of wide-scale eHealth implementation. Despite the fact that SSA experiences around 70% of all global HIV diagnoses, less than 23% of all HIV/AIDS research involves participation from researchers in Africa [1, 51]. Within the African continent, research tends to be concentrated in a few countries, predominantly South Africa, Uganda and Kenya [51]. There should be more HIV research in all countries across SSA, with more direct involvement of SSA researchers.

### Implications for Practice

The finding that eHealth interventions can improve behaviours related to HIV management in SSA is tremendously important. Through improving adherence to ART and retention in HIV care, eHealth interventions can be used to minimise the likelihood of people living with HIV transmitting HIV to others and reduce HIV-related morbidity and mortality across SSA [3, 52]. Healthcare systems across SSA face challenges with scare resources, inadequate finances and staff shortages, and low-cost eHealth strategies have huge potential to improve HIV-related outcomes across the region [53].

## Supplementary Information

Below is the link to the electronic supplementary material.

Supplementary file1 (DOCX 15 kb)

Supplementary file2 (DOCX 21 kb)

## Data Availability

No additional data available (common).
