# Supplementary material for: Effectiveness of eHealth Interventions for HIV Prevention and Management in Sub-Saharan Africa: Systematic Review and Meta-analyses
Source: AIDS Behav. 2021 Aug 24;26(2):457–69. doi: 10.1007/s10461-021-03402-w (PMC8813706; doi:10.1007/s10461-021-03402-w)
Supplement: Supplementary file 1 — Supplementary file1 (DOCX 15 kb) [file 10461_2021_3402_MOESM1_ESM.docx]

**Supplementary material 1**

**Search strategy for Medline, Embase, PsycInfo:**

1 HIV.tw.

2 human immunodeficiency virus.tw.

3 AIDS.tw.

4 acquired immunodeficiency syndrome.tw.

5 exp HIV/

6 exp HIV Infections/

7 sub-saharan africa.tw.

8 exp "Africa south of the Sahara"/

9 Angola.tw.

10 Botswana.tw.

11 Eswatini.tw.

12 Lesotho.tw.

13 Malawi.tw.

14 Mali.tw.

15 Mozambique.tw.

16 Namibia.tw.

17 South Africa.tw.

18 Zambia.tw.

19 Zimbabwe.tw.

20 Burundi.tw.

21 Eritrea.tw.

22 Ethiopia.tw.

23 Kenya.tw.

24 Rwanda.tw.

25 Somalia.tw.

26 South Sudan.tw.

27 Sudan.tw.

28 Tanzania.tw.

29 Uganda.tw.

30 Benin.tw.

31 Burkina Faso.tw.

32 Cabo Verde.tw.

33 Cameroon.tw.

34 Central African Republic.tw.

35 Chad.tw.

36 Comoros.tw.

37 Democratic Republic of Congo.tw.

38 Republic of Congo.tw.

39 Cote d'Ivoire.tw.

40 Equatorial Guinea.tw.

41 Gabon.tw.

42 Gambia.tw.

43 Ghana.tw.

44 Guinea.tw.

45 Guinea-Bissau.tw.

46 Liberia.tw.

47 Madagascar.tw.

48 Mauritania.tw.

49 Mauritius.tw.

50 Niger.tw.

51 Nigeria.tw.

52 "Sao Tome and Principe".tw.

53 Senegal.tw.

54 Seychelles.tw.

55 Sierra Leone.tw.

56 Togo.tw.

57 cell phon*.tw.

58 mobile phon*.tw.

59 cell telephon*.tw.

60 mobile telephon*.tw.

61 compute*.tw.

62 telemedicine.tw.

63 internet.tw.

64 online syste*.tw.

65 telecommunication.tw.

66 mobile applicatio*.tw.

67 communications media.tw.

68 computer communication networ*.tw.

69 mhealth.tw.

70 m-health.tw.

71 mobile health.tw.

72 ehealth.tw.

73 e-health.tw.

74 electronic health.tw.

75 educational technolog*.tw.

76 multimedia.tw.

77 social media.tw.

78 text messag*.tw.

79 SMS.tw.

80 vide*.tw.

81 smart phon*.tw.

82 smartphon*.tw.

83 digital media.tw.

84 digital health.tw.

85 digital health interventio*.tw.

86 digital interventio*.tw.

87 exp Telemedicine/

88 exp Computer Communication Networks/

89 exp Online Systems/

90 exp Communications Media/

91 exp Telecommunications/

92 Mobile Applications/

93 ICT.tw.

94 internet interventio*.tw.

95 internet-based interventio*.tw.

96 web-based interventio*.tw.

97 "information and communications technolog*".tw.

98 1 or 2 or 3 or 4 or 5 or 6

99 7 or 8 or 9 or 10 or 11 or 12 or 13 or 14 or 15 or 16 or 17 or 18 or 19 or 20 or 21 or 22 or 23 or 24 or 25 or 26 or 27 or 28 or 29 or 30 or 31 or 32 or 33 or 34 or 35 or 36 or 37 or 38 or 39 or 40 or 41 or 42 or 43 or 44 or 45 or 46 or 47 or 48 or 49 or 50 or 51 or 52 or 53 or 54 or 55 or 56

100 57 or 58 or 59 or 60 or 61 or 62 or 63 or 64 or 65 or 66 or 67 or 68 or 69 or 70 or 71 or 72 or 73 or 74 or 75 or 76 or 77 or 78 or 79 or 80 or 81 or 82 or 83 or 84 or 85 or 86 or 87 or 88 or 89 or 90 or 91 or 92 or 93 or 94 or 95 or 96 or 97

101 98 and 99 and 100

102 limit 101 to (english language and yr="2000 -Current")

**Search strategy for Web of Science and Cochrane library:**

Sub-Saharan Africa OR Angola OR Benin OR Botswana OR Burkina Faso OR Burundi OR Cabo Verde OR Cameroon OR Central African Republic OR Chad OR Comoros OR Democratic Republic of Congo OR Republic of Congo Or Cote d’Ivoire OR Equatorial Guinea OR Eritrea OR Eswatini OR Ethiopia OR Gabon OR Gambia OR Ghana OR Guinea OR Guinea-Bissau OR Kenya OR Lesotho OR Liberia OR Madagascar OR Malawi OR Mali OR Mauritania OR Mauritius OR Mozambique OR Namibia OR Niger OR Nigeria OR Rwanda OR Sao Tome and Principe OR Senegal OR Seychelles OR Sierra Leone OR Somalia OR South Africa OR South Sudan OR Sudan OR Tanzania OR Togo OR Uganda OR Zambia OR Zimbabwe in Title Abstract Keyword

**AND** HIV OR Human Immunodeficiency Virus OR AIDS OR Acquired Immunodeficiency Syndrome OR HIV infection in Title Abstract Keyword

**AND** cell phone OR mobile phone OR cell telephone OR mobile telephone OR computer OR telemedicine OR internet OR online systems OR telecommunication OR mobile applications OR communications media OR computer communication networks OR mhealth OR m-health OR mobile health OR ehealth OR e-health OR electronic health OR educational technology OR multimedia OR social media OR text message OR SMS OR video OR smartphone OR smart phone OR digital media OR digital health OR digital intervention OR digital health intervention OR ICT OR information and communications technology OR internet intervention OR internet-based intervention OR web-based intervention in Title Abstract Keyword

**Search strategy for Pan-African Clinical Trials Registry:**

Search terms: HIV

Recruitment status: Completed
