# Supplementary material for: Effectiveness of eHealth Interventions for HIV Prevention and Management in Sub-Saharan Africa: Systematic Review and Meta-analyses
Source: AIDS Behav. 2021 Aug 24;26(2):457–69. doi: 10.1007/s10461-021-03402-w (PMC8813706; doi:10.1007/s10461-021-03402-w)
Supplement: Supplementary file 2 — Supplementary file2 (DOCX 21 kb) [file 10461_2021_3402_MOESM2_ESM.docx]

**Supplementary material 2**

**Characteristics of the intervention and control arms:**

| Study | Arms of the trial |
| --- | --- |
| Barnabas 2016 | Intervention arm: SMS reminders were sent 3 weeks after testing for HIV with a follow-up phone call at 1 month. If men did not report to be circumcised at the 1-month follow-up call, a second SMS was sent 6-7 weeks after testing with a follow-up phone call at 2 months. Control arm: treatment as usual |
| De Tolly 2011 | Intervention arm: 3 informative SMS (sent 3 days apart). Intervention arm: 3 motivational SMS (sent 3 days apart). Intervention arm: 10 informative SMS (sent 3 days apart). Intervention arm: 10 motivational SMS (sent 3 days apart). Control arm: treatment as usual |
| Govender 2019 | Intervention arm: motivational SMS sent daily for the first week and then once a week thereafter. Control arm: treatment as usual |
| Haberer 2016 | Intervention arm: scheduled SMS medication reminders (every day for 1 month and then every week for the following 2 months). Intervention arm: triggered SMS medication reminders (2 hours after a missed dose). Control arm: treatment as usual |
| Harder 2019 | Intervention arm: motivational interviewing delivered via phone. Control arm: treatment as usual |
| Haruna 2018 | Intervention arm: sexual health game-based programme. Intervention arm: sexual health learning platform-based programme: Control arm: treatment as usual |
| Joseph Davey 2016 | Intervention arm: One-way SMS including reminders sent 1 week and 2 days before each appointment or medication pick-up and educational SMS sent every 60 days. Control arm: treatment as usual |
| Kalichman 2018 | Intervention arm: 5 counselling sessions on stigma and self-regulation delivered via mobile phone each week. Control arm: contact matched control |
| Kiwanuka 2018 | Intervention arm: short SMS and phone call visit reminders. Control arm: face-to-face visit reminders |
| Kurth 2019 | Intervention arm: internet-based interactive counselling programme delivered over 4 sessions. Control arm: treatment as usual |
| Lapinski 2008 | Intervention arm: film about HIV related social stigma. Control arm: treatment as usual |
| Leiby 2016 | Intervention arm: 21 conventional one-way SMS were sent over 6 months promoting voluntary medical male circumcision. Intervention arm: 21 tailored one-way SMS were sent over 6 months targeted at participants’ intention level to undergo circumcision. Control arm: treatment as usual |
| Lester 2010 | Intervention arm: two-way SMS each week and patients who responded with a problem were phone called by clinicians. Control arm: treatment as usual |
| Linnemayr 2017 | Intervention arm: one-way SMS. Intervention arm: two-way SMS. Control arm: treatment as usual |
| MacCarthy 2020 | Intervention arm: weekly SMS reporting participants adherence in the previous week. Intervention arm: weekly SMS reporting participants and peer’s adherence in the previous week. Control arm: treatment as usual |
| Mbuagbaw 2012 | Intervention arm: motivational SMS each week. Control arm: treatment as usual |
| Nsagha 2016 | Intervention arm: 4 SMS medication reminders each week for 4 weeks. Control arm: treatment as usual |
| Odeny 2014 | Intervention arm: one-way educational and reminder SMS messages sent once daily for the first week and then 7 further messages were sent over the following 35 days. Control arm: treatment as usual |
| Pop-Eleches 2011 | Intervention arm: short & daily text messages. Intervention arm: long & daily text messages. Intervention arm: short & weekly text messages. Intervention arm: long & weekly text messages. Control arm: treatment as usual |
| Reid 2017 | Intervention arm: one-way SMS reminding participants to pick-up their medication from the pharmacy 3 days and 1 day before and the morning of the pick-up. Control arm: treatment as usual |
| Van der Kop 2018 | Intervention arm: interactive SMS messages each week with follow-up phone calls if issues are raised. Control arm: treatment as usual |
| Venter 2019 | Intervention arm: app that provided HIV related information and appointment reminders. Control arm: treatment as usual |
| Winskell 2018 | Intervention arm: smartphone game to play for a minimum of 1 hour each day for a total of sixteen days. Control arm: treatment as usual |
| Ybarra 2013 | Intervention arm: internet based sexual health programme (5 sessions). Control arm: treatment as usual |
| Ybarra 2015 | Intervention arm: internet based sexual health programme (5 sessions). Intervention arm: internet based sexual health programme (5 sessions + review). Control arm: treatment as usual |
